# Health-oriented leadership in specialized outpatient palliative care teams in Germany: a qualitative study with palliative care professionals

**DOI:** 10.1186/s12904-025-01721-6

**Published:** 2025-03-28

**Authors:** Friederike Hess, André Klussmann, Volker Harth, Stefanie Mache

**Affiliations:** 1 Institute for Occupational and Maritime Medicine (ZfAM), University Medical Center Hamburg-Eppendorf (UKE), Seewartenstraße 10, Hamburg, 20459 Germany; 2 Department Health Sciences, Competence Centre Health (CCG), Hamburg University of Applied Sciences (HAW), Ulmenliet 20, Hamburg, 21033 Germany

**Keywords:** Health-oriented leadership, Palliative care, Healthcare, Leadership

## Abstract

**Background:**

Palliative care leadership is characterized by diversity and collaboration, with an emphasis on interdisciplinary teamwork and shared responsibility. In the context of palliative care, the leadership role is subject to a distinctive set of stressors, which in turn necessitates the implementation of strategies that are conducive to the well-being of the team. The implementation of the Health-oriented Leadership approach (HoL) appears to be a crucial step in advancing the field and addressing the emotional aspects of end-of-life care. Given the growing global need for palliative care, the study aims to provide initial insights into how health-oriented leadership is understood in the field of specialized outpatient palliative care (SAPV),its potential benefits and the challenges to its implementation. Furthermore, the perceived advantages of this approach in the given context will be discussed, and the challenges inherent to its implementation will be presented.

**Methods:**

In this qualitative study, a total of 30 semi-structured interviews were conducted with palliative care professionals working in Northern Germany. The participants were distributed equally between leaders and employees, and the interviews were conducted in person. The participants included medical practitioners, nursing staff and a health scientist in palliative care. The data were subjected to an inductive analysis and interpretation in accordance with the qualitative content analysis proposed by Mayring. Exploratory analyses were conducted to identify and examine the differences between leaders and employees.

**Results:**

The findings of the study indicate that employees and managers in palliative care hold comparable yet distinct perspectives on the advancement of occupational health (HoL). Both groups emphasised the relevance of a supportive working environment, health promotion measures and effective communication. Employees focused on work-life balance and immediate benefits such as job satisfaction, while managers prioritised adaptive structures and long-term goals such as reducing absenteeism. Communication barriers and resource management were identified as challenges. Both groups emphasised the importance of appreciation, mental health and professional boundaries for a healthy working environment.

**Conclusion:**

This study emphasizes the important role of health-oriented leadership in addressing unique challenges in SAPV and fostering a supportive work culture. It highlights the diverse approaches of leaders and employees towards health management and underscored the increasing emphasis on self-care and mental well-being in high-stress healthcare environments.

**Supplementary Information:**

The online version contains supplementary material available at 10.1186/s12904-025-01721-6.

## Introduction

Those engaged in the provision of specialised outpatient palliative care (SAPV) are confronted with a range of significant professional challenges [1]. The care of seriously ill and dying patients, the interaction with grieving families and the demonstration of a high degree of empathy can result in emotional exhaustion [1, 2]. The physical strain caused by irregular working hours, on-call duties and long journeys between patients, as well as strenuous nursing tasks, has a negative impact on well-being. The high number of patients, extensive administrative tasks and limited resources result in increased time pressure and stress. Furthermore, management and care problems, as well as a continuous need for further training, lead to an increase in the stress situation for employees in SAPV [1, 3–5].

Given the numerous challenging stressors encountered by palliative care (PC) professionals, it is of paramount importance to implement leadership strategies that effectively maintain and enhance the overall well-being of the interdisciplinary team [6, 7]. Leadership in palliative care is a multifaceted concept that encompasses various domains, including clinical practice, research, policy and advocacy, and education [8]. The presence of collaborative, interdisciplinary leadership may be a prerequisite for advancing palliative care and facilitating successful healthcare transformation. An essential quality of effective leaders is the capacity to empathise with the needs of their staff [9]. Effective leadership can be a vital component in optimising palliative care. The foundation for excellent patient support is laid by strategic resource allocation, staff development and the assurance of high-quality care [10–12]. Furthermore, a supportive work environment has been demonstrated to have a significant positive impact on the satisfaction and well-being of nursing staff, which in turn as a beneficial effect on the quality of patient care [10, 13].

It is proposed that health-oriented leadership (HOL) could play a pivotal role in this context. Franziska Franke and Joerg Felfe have developed this approach based on the principles of transformational leadership. It employs a systematic examination of the health behaviours of both leaders and followers, thereby enabling inferences to be drawn about their interrelationships [14, 15]. To date, there has been no empirical research investigating the perception of a health-oriented management style in the context of specialised outpatient palliative care.

The conceptualization and perception of a health-oriented leadership style in the field of specialized outpatient palliative care has not yet been explored. However, research into health-orientated leadership in palliative care is of great importance for several reasons.

The implementation of strategies that prioritise a health-centred approach to leadership could prove an effective means of alleviating the stress experienced by those engaged in palliative care. This can be achieved through the prioritisation of support, appreciation and the promotion of self-care [16]. Health-oriented leadership fosters a culture of open communication, respectful interaction, and mutual support, thereby enhancing teamwork [14, 17]. This is of particular importance in the context of palliative care, where interdisciplinary collaboration is of paramount importance. Health-centred leadership can play a preventative role by promoting healthy working practices and creating a supportive working environment that protects the mental health of employees [18]. A healthy and supportive work environment has been demonstrated to have a positive impact on employees, as well as on the quality of patient care [19, 20]. The presence of healthy and motivated employees within a healthcare setting enables them to work empathetically and competently, which in turn may result in enhanced patient satisfaction and more favourable treatment outcomes [21, 22]. It can be reasonably deduced that research into this topic would prove beneficial in improving both the working conditions of employees and the quality of care, which would be of great value to the entire healthcare system.

### Study aim

Therefore, this study aims to provide a preliminary understanding of health-oriented leadership within the SAPV sector, while equally considering the perspectives of both employees and leaders.

The study focuses on the following research questions:

1. *What is the current conceptualization of health-oriented leadership among leaders and employees in specialized outpatient palliative care teams?*
2. *What are potential benefits of a health-oriented leadership approach?*
3. *Which factors can challenge the implementation of health-oriented leadership?*

## Methods

### Study design

Given the unexplored nature of the research area, a qualitative research methodology was adopted. Therefore, the study involved a primary data collection process. Semi-structured, guided interviews were conducted to capture the subjective perceptions of individuals within the SAPV sector. In addition, a comparison of perspectives between managers and staff was sought. The interviews were conducted as 'face-to-face' conversations between the interviewer and the interviewee. The interviewees had received the informed consent and data protection forms in advance by e-mail. In addition to the interviews, a short socio-demographic questionnaire was used. Pre-tests were carried out prior to data collection to ensure ease of use. Content of the interview guides can be found as Supplement 2.

### Ethical considerations

The study was conducted in accordance with the Declaration of Helsinki and approved by the Ethics Committee of the University Medical Center Hamburg-Eppendorf, Germany—LPEK-0690.

### Study participants

The study focused on the professional group of palliative care workers and their managers in the context of specialised outpatient palliative care services. The aim was to interview both medical and non-medical staff in order to gain interdisciplinary insights into the topic. In order to gain a comprehensive understanding of the subject matter, it was essential to consider the perspectives of a diverse range of employees in palliative care. Furthermore, it was imperative to incorporate the perspectives of both employees and managers, which is why the study involved a diverse range of participant groups.

Interviewees had to have at least one year's experience in palliative care and work at least 20 h per week in the SAPV sector to be eligible for this study. Nurses were required to have specialist training in palliative care [23]. Doctors should have advanced training in palliative medicine, known as 'additional training in palliative medicine for doctors' [23, 24]. Leaders were expected to have a health-related background, such as nursing or health sciences. Respondents had to have at least one year's experience in palliative care and work at least 20 h per week in SAPV to be eligible for this study.

### Recruitment

Participants were selected via convenience sampling. With the approval of the board of directors, the interviewer sent emails to palliative care cooperatives in Germany with information about the study and an invitation to participate. Two weeks later, after the first participants had been recruited, a second email was sent to the interviewees. Participation in the study was voluntary, and the anonymity of the participants was maintained by the interviewer throughout the process.

### Sample size

In order to achieve a congruent result within the chosen research methodology, the number of interviews was set at thirty. It is expected that theoretical saturation will be reached at this point. Theoretical saturation is reached when no new relevant themes or variations emerged during the final interviews and data analyses that extended the understanding of the phenomena under investigation [25]. Data analysis was continuous throughout the investigation, and we observed a recurrence of the same concepts that had emerged in previous interviews. The research team regularly reviewed the data and the emergence of patterns to ensure that all relevant dimensions were covered. We found that no new, significant information was added, which confirmed the saturation.

The aim is to gather enough comprehensive information to develop and understand the core categories and concepts relevant to the purpose of the study. It is expected that the data collected will be sufficient to support the creation of a well-founded theoretical framework to encourage further research [26].

### Data collection

The interviews took place between August 2023 and February 2024. The interviews were conducted at the respondents' place of work. The interview lasted between 10 and 25 min, with an average of 16.75 min. Only the interviewer (FH) and the interviewee were present throughout the interviews. The female interviewer holds a Master of Science degree in Public Health and has acquired the requisite experience to conduct interviews. Two separate interview guides were used to interview managers and employees. The manager guide consisted of six main questions with several optional sub-questions.

The employee guide consisted of seven main questions with several optional sub-questions (see supplement 2). Six of the main questions were the same for both groups. One question was specific to the staff. All interviews were translated from German into English.

The audio files of all interviews were pseudonymised and will be stored at the Institute of Occupational and Maritime Medicine for ten years.

### Data analysis

Based on the transcripts, inductive categories were formed and the texts were coded (see Table 1). The software MAXQDA Plus 2022 Semester (2023) was used for transcription, coding and analysis.

**Table 1:** Interview-categories and sub-categories

Theme	Category	Subcategories
**Conceptualization of Health-Oriented Leadership in SAPV: Understanding and Measures**	***Holistic approach ***	*Health as a holistic management approach that takes into account both professional performance and the personal well-being and circumstances of employees*
	***Anchoring health orientation in the corporate culture***	*Health orientation as an integral part of corporate culture and management ethics*
		*Commitment of the highest management level to maintaining employee health*
	***Egalitarian and respectful communication***	*Need for respectful communication across all hierarchical levels*
		*Promoting a non-hierarchical working environment that favours effective teamwork and a healthy working atmosphere*
	***Importance of support and trust***	*Access to support for difficult patients or stressful circumstances*
		*Establishing open and trusting communication*
	***Boundaries between work and private life***	*Ensuring clear working hours, respected breaks and the avoidance of overwork and burnout by recognising symptoms of stress and overwork*
		*Compliance with working hours and rest periods, e.g. between early and late shifts*
	***Preventive measures and safety***	*Preventive health and safety measures in the workplace to avoid accidents or harmful behaviour*
		*Proactive measures to reduce health risks in the working environment*
	***Customisation of working conditions***	*Flexibility in working hours and support for employees with health or private concerns*
		*Adaptation of working conditions to the individual needs of employees (e.g. ergonomic furniture)*
**Potential benefits**	***Increase employee motivation and satisfaction***	*Promoting a feeling of appreciation and satisfaction among employees*
		*Reduction of individual stress levels and coping with stress through health-oriented leadership*
	***Promotion of team cohesion and cooperation***	*Improving the working atmosphere and Promoting team harmony*
		*Supporting an open and respectful dialogue within the team*
	***Reduction in sickness absence***	*Potential reduction in days of absence due to illness*
		*Better health and higher employee motivation through health-orientated management*
	***Building trust and loyalty to the company***	*Creating trust through health-orientated leadership*
		*Strengthening employee loyalty and identification with the team and company*
	***Employee retention and avoidance of skills shortages***	*Increase employee retention through a health-promoting work environment*
		*Prevention of long-term illnesses through a better understanding of health in the working environment*
	***Promotion of mental health and collegial support***	*Supporting mental health and promoting mutual support within the team*
		*Improving social interaction and collaboration by promoting mental health*
**Challenges for the implementation of health-oriented leadership**	***Balance between economic efficiency and health***	*Economic requirements vs. health promotion*
		*Need for a balance between profit-orientation and health protection in the workplace*
	***Communication and support***	*Importance of open communication in relation to health issues*
		*Support from managers, especially for mental health issues*
		*Effects of a lack of communication on conflicts and the working environment*
	***Time management and work pressure***	*Lack of time as a challenge for health-orientated discussions and meetings*
		*Stress caused by high work density and its influence on the prioritisation of health initiatives*
	***Voluntariness vs. coercion in supervision***	*Discussion about voluntary vs. mandatory participation in supervision*
		*Challenges in integrating supervision into everyday working life*
	***Integration of technology***	*Technical requirements and training on the use of digitalisation*
		*Burden due to additional training requirements for dealing with technology*
	***Logistical hurdles and participation in health programmes***	*Logistical problems when planning health programmes (e.g. employee involvement, schedules)*
		*Low participation due to lack of time or disinterest*
	***Building trust in the team***	*Need to build trust within the team for successful health promotion*

Following the encoding of the initial interview, the results were discussed by the research team. Thereafter, the defined subcategories and allocation of text segments were compared until a consensus was reached. The interviews were encoded by two researchers. In the event of discrepancies or uncertainties regarding text passages or codes, discussions were held during regular project meetings. To guarantee a systematic and transparent data analysis, an inductive approach was selected, aligning with Mayring's qualitative content analysis (controlled analysis of fixed communication guided by content analytical rules and categories following a stepwise approach, without quantifying results).

To illustrate the results, quotations from participants were translated into English.

### Criteria for assessing qualitative research

We addressed the following quality criteria for our study: 1. Procedural rigour (i.e. consistency, transparency etc.); 2. Representativeness; 3. Clarification and justification; 4. Interpretative rigour; 5. Reflexivity; and 6. Transferability (see Table 2).

**Table 2:** Criteria for assessing qualitative research

Rigour aspect	Description of how it was addressed	Examples
Credibility	Ensure that the results truly reflect the participants' perspectives and do not contain any bias from the researcher or other external factors	Techniques such as ‘member checks’ (participants checking that the results accurately reflect their perspectives) were used to validate the results
Dependability/ Consistency	Ensure that the research process is consistent and comprehensible	An ‘audit trail’ was kept in which all decisions and changes in the research process were documented. Standardised procedures for data collection and analysis were also used to ensure consistency
Clarification	Ensure that the interviewee's answer is understood correctly and completely. It helps to eliminate ambiguities and obtain more details that make the answer more precise	Interview techniques were used: the interviewer asked specific follow-up questions if something is unclear or if the answer is too vague Comprehension check: The interviewer summarises the interviewee's answer in their own words and asks whether this summary is correct
Justification	Understanding the reasons or motives for a particular decision, action or opinion. This helps to understand the interviewee's way of thinking and decision-making processes	Interview techniques were used: Asking about the reasons and considerations behind a decision or action, e.g. the interviewer asked for the reasons why the interviewee acted in this way or made a certain decision
Confirmability	Demonstrate that the results are based on the data collected and were not influenced by the researcher's biases or subjective opinions	A log of decisions and reflections (audit trail) was kept minimising the influence of the researcher. In addition, the data was compared with other researchers to minimise bias
Authenticity	Ensure that the participants' perspectives are reflected authentically and without distortion	An ‘open coding’ approach was used to ensure that all participants' perspectives were considered equally. Care was also taken to ensure that no interpretation of data by the researcher was included in the analysis
Reflexivity	The researcher regularly reflects on their own influence on the research process and recognises their own biases and perspectives	The researchers regularly documented their own assumptions and influences in reflections to show how these might have influenced the data and analysis. For example, a detailed reflection on their own preconceptions about the research topic was created
Transferability	Demonstrate that the results can be applied to other contexts or populations	Detailed contextual information (e.g. demographic data characteristics) was provided to enable other researchers to transfer the results to their own context

Moreover, the COREQ Checklist (Consolidated criteria for reporting qualitative research) was used as a reference for reporting the results of the study (see the supplement 1).

## Results

### Socio-demographic data

The sample size of *n* = 30 interviewees was comprised of 15 staff members and 15 leaders. The interviewees were aged between 25 and 64 years. Of the 30 specialists in palliative care and physicians from Northern Germany who were interviewed, 76% were female and 90.1% were employed on a full-time basis, with a range of work experience from one to over ten years at the time of the survey. The majority of the interviewees were qualified as specialists in palliative care (54.5%) or as specialist physicians in this field (38.9%). More detailed information can be found in Table 3.

**Table 3:** Sociodemographic characteristics of participants (*n* = 30)

	*n*	%
Gender		
Male	7	66
Female	23	34
Diverse	0	0
Age		
≤ 24 years	0	0
25–29 years	2	6
30–34 years	3	12
35–39 years	4	15
40–44 years	7	22
45–49 years	5	16
50–54 years	4	14
55–59 years	3	9
≥ 60 years	2	6
Type of employment		
Temporary contract	4	10
Permanent contract	27	90
Part-time	4	10
Full-time	27	90
Highest educational level		
General secondary school	0	0
Intermediate secondary school	17	57
Specialised grammar school	0	0
Grammar School	0	0
High school	13	43
Professional qualification		
Carer (with specialist training)	16	53
Carer (without specialist training)	0	0
Nursing assistant	0	0
Physician	11	37
Psychologist	2	7
Other	1	3

### Conceptualization of health-oriented leadership in SAPV

The employees' comprehension of health-oriented leadership within SAPV underscored the significance of egalitarian and respectful communication across all professional levels. The strategy was anticipated to foster a non-hierarchical work environment, thereby facilitating effective teamwork and a salutary working atmosphere. They held the conviction that each role in palliative care was indispensable, and that mutual respect and appreciation were indispensable.

*“In the palliative care sector, each professional group is vitally important. Moreover, every group must communicate with the others. It's essential to be informed of everyone else's status as well. After all, the focus is on the patient. We, as professional groups, revolve around this central concern. That's why communication and teamwork are so crucial.” (Audio 11.MP4, Pos. 5)*

*“Through the necessity of always working together with all professional groups and within the team. Simply to ensure healthy leadership or a healthy work environment, yes, I would actually describe it as a headline in some way: At eye level.” (Audio 11.MP4, Pos. 3)*

Moreover, employees indicated that access to support was crucial when confronted with challenging patients or circumstances, and that they needed to feel empowered to seek assistance. Leaders were perceived as instrumental in establishing a culture of open and trusting communication, where employees could engage in constructive dialogue about their experiences without fear of reprisal.

*“It’s important that employees know whom to contact when they encounter issues with challenging patients or in the event of a particularly distressing death.” (Audio 9.MP4, Pos. 5)*

Furthermore, the respondents identified the maintenance of clear boundaries between work and personal life, the assurance of predictable working hours, the respect of designated service and rest times, and the avoidance of burnout through the recognition of stress and overwork indicators as essential factors.

*“[…] that the employer ensures that working hours are adhered to. That rest periods between late and early shifts, for example, are maintained. That one does not have to work again immediately after being on call, for example.” (Audio 9.MP4, Pos. 3)*

*“Health-oriented leadership would entail administrative supervisors recognizing issues such as overload, burnout, and the like, and taking steps to prevent such issues.” (Audio 7.MP4, Pos. 7)*

In conclusion, the concept of health-oriented leadership was interpreted by employees as a strategy that guarantees the well-being of employees through the implementation of preventive and safety measures, and the establishment of a supportive and caring environment that facilitates professional growth and personal health maintenance.

*“It is important that leadership contemplates the measures necessary for health prevention, or prophylaxis, and also considers safety measures at work to prevent accidents or behaviors that could potentially result in harm to the employee.” (Audio 2.MP4, Pos. 3)*

In response to the question regarding their comprehension of health-oriented leadership, the leaders placed significant emphasis on the establishment of structures that could be tailored to the personalities and life circumstances of employees. This encompassed flexible working hours and assistance in the event of accidents or health-related concerns.

*“What can I contribute as a company, as a leader, to reduce the health-related strain in the job as much as possible? And what options do I have to possibly promote health in the workplace and customize it to meet the individual needs of the employees?” (Audio 6.MP4, Pos. 3)*

From the perspective of those in leadership roles, it was considered crucial to guarantee that the work environment did not have a detrimental effect on the health of employees, which was seen as an integral aspect of health-oriented leadership.

*“What can actually affect health? And I would say it depends on a variety of things. Not just the working hours here, when people work in the office, but that they have ergonomic furniture, that it is properly adjusted. That breaks are adhered to. So all occupational safety measures, that's all actually health.” (Audio 3.MP4 FK, Pos. 19).*

Furthermore, it was acknowledged that while it was not possible for leaders to alter personal issues affecting their employees, they could nevertheless proactively establish a supportive environment. It was recognized that the private nature of employees' health challenges was of significance, and thus leaders designated health-oriented leadership as a holistic approach, considering not only the performance and comfort of employees in their roles, but also their personal well-being and life situations.

*“Health-oriented leadership means holistic management. That is, I don't just look at how well colleagues do their work and how comfortable they feel in it. But it's also a bit more about, so to speak, focusing on the structures, […].” (Audio 12.MP4, Pos. 3)*

In essence, health-oriented leadership may be defined as the creation of a corporate culture wherein the concept of health is regarded as an intrinsic and organic element of the organizational ethos. The respondents indicated that health-oriented leadership was a value that needed to be embedded in the organisation's culture and that the willingness to maintain the health of employees must be present at the highest level of management.

*“And then, I believe, there must be a commitment from the highest level to maintain the health of the people or to manage the team effectively. It's about having a good team, about having motivated individuals.” (Audio 12.MP4 FK, Pos. 29)*

#### Potential benefits of health-oriented leadership

In their interviews, employees indicated that health-oriented leadership could confer significant advantages with respect to healthcare. The approach fostered a sense of value and satisfaction among the staff, which contributed to the creation of a positive working atmosphere.

*“Definitely much more satisfaction and a sense of appreciation. Perhaps also that one does not feel so burdened oneself. We all know that [our] profession, or the health profession, is a very stressful one.” (Audio 11.MP4, Pos. 13)*

Moreover, the implementation of a health-oriented leadership approach could facilitate the promotion of team harmony and collaboration. Such an approach would foster a supportive environment in which employees feel acknowledged and appreciated, which in turn could enhance retention and commitment.

*“Additionally, it's about shaping the atmosphere, including the direct ways of interacting within the team, the conversations. And I’m not talking about the supervision, but rather about fostering a work environment where exchange is possible, where such exchange is appreciated, and not perceived as a weakness when one asks questions and seeks advice within the team.” (Audio 10.MP4, Pos. 17)*

It was also suggested that this could result in a reduction in the number of sick days taken by employees, which would contribute to a healthier and more engaged workforce.

*“It can reduce the number of sick days if done correctly. And then people are more likely to stay in their job and not leave, or they won't retreat into internal migration or be absent all the time.” (Audio 7.MP4, Pos. 21)*

It is noteworthy that the interviewed employees indicated that they were able to establish trust in their employers under leadership that prioritized health and wellbeing. This was perceived to foster a stronger sense of connection and loyalty to the team and the organization.

*“Yes, it has only a positive impact, really. For one, it's knowing that my employer feels a sense of responsibility and offers support. This initially gives me confidence that someone is watching out for me, providing a measure of security. Naturally, this enhances my connection with the employer, and by extension, the whole team, right? So, when I feel well-cared-for and supported, I find that I identify more with the team and so on. […]” (Audio 2.MP4, Pos. 15)*

The concept of health-oriented leadership, as elucidated by numerous leaders during the course of their interviews, was found to exert a beneficial influence, resulting in a multitude of tangible benefits within the workplace. In view of the acknowledged scarcity of proficient personnel in the field, this leadership strategy may prove instrumental in the retention of employees. It was posited by the leaders that the instillation of a clear and comprehensive understanding of health would result in employees deriving greater enjoyment from their work environment, which would in turn foster greater employee retention.

“*The influence is, of course, that it’s particularly beneficial in the context of the skilled labor shortage. It prevents situations leading to long-term illness from arising in the first place. Instead, it ensures that people have a clear understanding of what health means. Precisely, I believe this can foster employee retention by making sure they are happy to work here.” (Audio 3.MP4 FK, Pos. 39)*

Furthermore, the leaders observed a potential reduction in employee sickness rates following the implementation of health-oriented practices. The reduction in absenteeism due to illness was considered to be directly correlated with enhanced motivation among team members, which in turn led to a consistent and efficient work output.

*“[…] The rate of sickness can be reduced if people execute it well, leading to an increase in the motivation of colleagues to remain with the company. This in turn cultivates greater loyalty and dependability towards the employer, resulting in satisfaction, personal satisfaction.” (Audio 6.MP4 FK, Pos. 11)*

Furthermore, interviewees posited that health-oriented leadership could foster an environment of collective support and care for mental health. The leaders observed that employees who were in a position of good mental health were better able to offer support to their colleagues, thereby fostering a collaborative and supportive atmosphere within the team.

*“Because I believe it is simply a good tool, and also for people who say: 'I personally am fine. I don't need the supervision.' But supervision also thrives on people who have good approaches to maintaining their mental health, helping others in doing so, ensuring that there's mutual support.” (Audio 4.MP4 FK, Pos. 21)*

#### Challenges for the implementation of health-oriented leadership

It was acknowledged by employees that the implementation of health-oriented leadership required a careful balancing act between economic viability and the integration of health-focused practices.

*“[…] there is this balance between business economic thinking and maintaining health at work. Naturally, one must think economically; you need to generate profits to be able to make things happen. However, if everything is driven solely by business management criteria, the result is that employees, unfortunately, end up being processed through the system.” (Audio 10.MP4, Pos. 21)*

It was asserted that enhanced communication and more robust support structures are required within the teams, particularly with regard to sensitive matters such as mental health. The absence of open dialogue and adequate support from leadership could precipitate internal conflicts and impede the establishment of a health-focused working environment.

*“And if it seems like there's always time pressure and a constant rush of “We need to get to the next appointment”, then naturally, the barrier to initiate a conversation with someone is much higher, right?” (Audio 2.MP4, Pos.23)*

Furthermore, the limited time available presented a challenge for employees in terms of identifying suitable locations for essential health-oriented discussions, supervision sessions, and meetings. Such time constraints could contribute to employee burnout, thereby rendering it challenging to foster and maintain a health-oriented leadership approach.

*“The challenge is always the same, (laughs) yes, it's much like it is everywhere in healthcare: the factor of time. […] That's what can kill everything. The fact that there is so much work that you don't know how to accommodate it all. And then, as a result, it automatically gets deprioritized. It's essentially always the same mechanism.” (Audio 2.MP4, Pos. 25)*

*“So, the top priority is simply the care of our people[patients], I believe. And if I think that I have a supervision here and another meeting there, and this and that. It all has to fit within the working hours.” (Audio 8.MP4, Pos. 31)*

Those in leadership roles in SAPV have indicated that they were responsible for striking a delicate balance between mandatory and voluntary participation in supervision, which is of critical importance for the implementation of health-oriented leadership.

*“We have given a lot of thought to whether to make it compulsory, but I’m not in favor of that because I believe that if you attend supervision sessions reluctantly, they won’t be beneficial to you. You won’t be open to the process, nor will you embrace it to any significant degree.” (Audio 6.MP4 FK, Pos. 16)*

During the interviews, it was noted that the integration of technology, while maintaining patient care standards, required substantial training and time, which further increased the already considerable workload.

*“A challenge, for instance, is technical equipment. While digitalization is indeed beneficial in reducing bureaucracy and streamlining work, it also demands considerable time for training so that people are able to use it effectively.” (Audio 3.MP4 FK, Pos. 63)*

In the context of the daily pressures inherent to the role, those in positions of leadership were acutely aware that health initiatives could easily be marginalized. It is possible that employee participation may decline as a result of a lack of interest or the perception that these programs are onerous. The implementation of health programs may be further complicated by logistical hurdles, such as the coordination of staff availability and the scheduling of activities around tight work commitments.

*“Because there are many who simply go home and do nothing, which isn't exactly health-promoting. Therefore, I believe if the system is better organized again, so that the schedules are more relaxed, we could reintroduce this concept to our colleagues, suggesting that it could have a positive impact.” (Audio 5.MP4 FK, Pos. 21)*

Furthermore, the necessity of fostering a relationship based on trust with the team members was emphasized. The internal dynamics of the group and the requirement for trust-building introduced additional complexities, as they sought to engage all team members and bridge the gap between the managerial objectives and the reality of daily work.

*“We are essentially caught in a vicious cycle. A high degree of communication ability and empathy is crucial, I believe, so that you can be more attuned to and connect better with your colleagues. These, in my opinion, are the key factors that enable health-oriented leadership to be effective. It doesn't work when done in passing.” (Audio 5.MP4 FK, Pos. 27)*

## Discussion

The objective of this study was to examine the concept of health-oriented leadership in specialist outpatient palliative care, which had not previously been the subject of investigation. The study aimed to provide an initial overview of how leaders and staff in the sector understand health-centred leadership, the challenges to its implementation and its potential benefits.

### Understanding of a health-oriented leadership style in SAPV

In this study, both employees and leaders demonstrated a shared understanding of health-oriented leadership, although their perspectives on this concept differed to some extent. Both groups concurred on the importance of a supportive work environment, proactive health management, and effective communication and teamwork. These findings are corroborated by the results of Dahlin et al. (2019) and Hewison et al. (2019). The former studies emphasised the pivotal role of a supportive work environment in enhancing the satisfaction and well-being of nursing staff, which in turn positively impacts patient care. While employees in this study concentrated on aspects such as work-life balance and the implementation of structured support systems, leaders focused on the creation of adaptive structures and the embedding of health values within the organisation's culture. Despite their differing foci, their perspectives were complementary, combining to form a comprehensive approach to health-oriented leadership in the sector [8, 9].

The behavioural dimension, which pertains to the extent of personal involvement in health-related actions and behaviours, was discernible in the study. The importance of a supportive work environment and proactive health management was acknowledged by both employees and leaders in SAPV. This was particularly evident in the emphasis placed by employees on the importance of work-life balance, and the focus of leaders on the creation of structures that facilitate the promotion of health behaviours [15].

The study identified a number of challenges faced by employees and leaders in the implementation of health-oriented leadership, including communication difficulties and administrative issues. These findings are reinforced by those of Baqeas et al. and Maffoni et al., who highlighted the stressors faced by professionals in palliative care and the need for effective leadership strategies to support the well-being of the interdisciplinary team [6, 7]. Moreover, the findings align with those of Kauffmann et al., which highlighted the impact of work organisation and overload on healthcare staff, particularly in emotionally demanding fields like palliative care [27]. This is evidenced by the concerns expressed by employees regarding communication barriers and time management. The focus of leaders on administrative challenges has been shown to resonate with the broader issue of resource allocation and quality management in palliative care [10–12]. In order to address these challenges, it is necessary to expand the provision of services and to improve the coordination of information [28, 29]. Both groups demonstrated a commitment to the effectiveness of health-oriented leadership. The employees questioned how these practices were to be integrated into their daily work routines, while the leaders considered the implementation and sustainability of these practices within the context of organisational constraints.

The aforementioned immediate benefits, as identified by employees, are of paramount importance in a sector where the satisfaction of nursing staff can have a significant impact on patient care [10, 13]. The focus of leaders on long-term strategic benefits, such as the reduction of absenteeism and the enhancement of staff retention, was aligned with the necessity of developing staff and ensuring the provision of high-quality care in the context of the increasing demand for palliative services in Germany [30–32].

In conclusion, the perspectives of employees and leaders on health-oriented leadership, although occasionally divergent in emphasis, were mutually reinforcing. They combined immediate, practical benefits with long-term strategic advantages, thereby underscoring the multifaceted nature of this approach in the context of palliative care. The emphasis on shared leadership and the importance of interdisciplinary teams is consistent with the insights of Dahlin et al., who discussed the various aspects of palliative care leadership [8].

### Strengths and limitations

In conducting this research study on health-oriented leadership in specialised outpatient palliative care, a qualitative methodology was selected as the most appropriate approach for an exploratory investigation into a field with limited prior research. This approach proved conducive to the capture of the subjective experiences and perceptions of individuals operating within this sector. In this study, we put forward relevance, validity and reflexivity as the overarching standards for our qualitative inquiry: we used relevance as an important criterion for the selection of the research topic as well as the research questions. The choice of topic—the perception of a health-oriented leadership style in specialised outpatient palliative care—is particularly relevant as this area has little empirical research but is central to improving the quality of care and the well-being of staff. To further emphasise the relevance, we have looked closely at the context and specific challenges of specialised outpatient palliative care and how leadership in this area can influence the success of care facilities. This contributes to the practical significance of the study and creates a link to the current debate on the importance of health-centred leadership in healthcare.

Validity in this case refers to how well the chosen method and data reflect the actual perspectives and experiences of the participants. To ensure validity, we used several techniques, such as member checking, in which participants reviewed the results to ensure that their perspectives were accurately captured. Reflexivity played a central role in the entire study. As qualitative research is always influenced by the perspectives and background of the researcher, we systematically reflected on our own role and knowledge in the research process. This was supported by regular reflection logs in which we documented our own assumptions, biases and the influence of my perspective on the data analysis. These reflections not only helped us to recognise potential biases, but also helped to make the interpretation of the results more transparent and understandable. Explicitly emphasising reflexivity in the article helps readers to better understand the process of knowledge generation and to have confidence in the independence and objectivity of the analysis. By combining these three concepts—relevance, validity and reflexivity – we were able to ensure that our study is not only scientifically sound but also practically applicable. This approach helps to ensure that the findings are relevant to both the research community and palliative care practitioners, and that the research processes are transparent and accountable.

The decision to include both leaders and employees in the SAPV sector was made deliberately in order to ensure a broad perspective. The participants, who were drawn from a variety of professional backgrounds, including medicine, nursing and health science, contributed a wealth of diverse perspectives to the data set. It was anticipated that this approach would yield sufficient, valid and reliable data.

It should be noted, however, that the study's design was not without certain limitations. While the sample size of interviewees was adequate for qualitative research, it may not fully represent the broader spectrum of experiences within the SAPV sector. This limitation is intrinsic to qualitative research, which typically prioritises in-depth analysis over extensive scope. Nevertheless, the study yielded a considerable amount of data. To ensure clarity and relevance, only the data most pertinent to the research questions could be included. This selective approach permitted a concentrated and in-depth examination of the key issues, despite the wealth of information available. It is conceivable that the recruitment strategy employed may have resulted in the introduction of a selection bias, as participants were predominantly recruited through social networks, specific organisations, and personal contacts. This may have resulted in certain social or demographic groups being overrepresented in our sample. To address this bias, we utilised various recruitment sources to ensure the most diverse sample possible and to target underrepresented groups. In addition, we regularly reflected on the recruitment process to ensure that different.

### Research implication and practical implication

The findings of this study have the potential to inform both practice and policy in a number of significant ways. By identifying leadership practices that could promote mental health and resilience among staff, this research may inform the structuring of management strategies within palliative care organizations. The implementation of health-oriented leadership practices has the potential to reduce burnout and stress, which may subsequently lead to an improvement in job satisfaction and motivation among caregivers.

Furthermore, the findings could inform training programs for current and future leaders in palliative care, equipping them with the necessary skills to foster a supportive work environment. Policymakers might also find this research useful for developing guidelines and standards for leadership practices in healthcare settings, potentially promoting a more sustainable and healthier workforce. By addressing the emotional and physical demands on palliative care workers, health-oriented leadership could benefit both employees and patient outcomes, thereby contributing to the overall efficacy and sustainability of palliative care services.

## Conclusion

This study contributes to the emerging field of health-oriented leadership within the context of specialized outpatient palliative care. These study results underscore the pivotal role of leadership and proactive mental health initiatives in safeguarding the well-being of these professionals and, subsequently, the quality of care they provide. The findings indicate a consensus among employees and leaders regarding the significance of health-oriented leadership. Furthermore, the study highlights the pivotal role of a supportive work environment in enabling a health-oriented leadership approach and elucidates the intrinsic complexities of adopting health-oriented practices within a healthcare context.

In sum, the findings provide insights that could inform future research and guide the implementation of health-oriented leadership approaches in these settings.

## Supplementary Information

Supplementary Material 1.

Supplementary Material 2.

## Data Availability

The datasets generated and analysed during the current study are not publicly available due to German national data protection regulations. They are available from the corresponding author on reasonable request.

## Abbreviations

HoL: Health-oriented leadership
SAPV: Specialized outpatient palliative care
PC: Palliative care
ZfAM: Zentralinstitut für Arbeitsmedizin und Maritime Medizin/ Institute for Occupational and Maritime Medicine
