# Supplementary material for: Health-oriented leadership in specialized outpatient palliative care teams in Germany: a qualitative study with palliative care professionals
Source: BMC Palliat Care. 2025 Mar 28;24:84. doi: 10.1186/s12904-025-01721-6 (PMC11951508; doi:10.1186/s12904-025-01721-6)
Supplement: Supplementary file 2 — Supplementary Material 2. [file 12904_2025_1721_MOESM2_ESM.docx]

# Supplement 2: Interview Guide

Leaders

| No. | Main Question | Additional Question |
| --- | --- | --- |
| 1 | What do you understand by health-oriented leadership? | 1. What role do you think it plays in SAPV? |
| 2 | Does your current employer offer any health promoting measures? | 1. Which ones for example? 2. Do you use these measures? 3. If not, what do you think is the reason for this? |
| 3 | What influence, in your opinion, can health-oriented leadership have on the team? | 1. Would you say that health-oriented leadership adds value to the SAPV sector? |
| 4 | What do you think is needed for changes in the health of team members to be noticed in time? |  |
| 5 | What conditions can facilitate the implementation of health-oriented leadership? | 1. What challenges are there? |
| 6 | Do you have any further ideas or suggestions for effectively implementing health-oriented leadership in outpatient palliative care? | 1. What would these be?   Can you share your ideas with the management team? |

Employees

| No. | Main Question | Additional Question |
| --- | --- | --- |
| 1 | What do you understand by health-oriented leadership? | 1. What role do you think it plays in SAPV? |
| 2 | Does your current employer offer any health promoting measures? | 1. Which ones for example? 2. Do you use these measures? 3. If not, what do you think is the reason for this? |
| 3 | What influence, in your opinion, can health-oriented leadership have on the team? | 1. Would you say that health-oriented leadership adds value to the SAPV sector? |
| 4 | What role does mental health play in SAPV? | 1. Do you believe that health-oriented leadership can contribute to the mental health of the staff? 2. If yes, how? 3. If not, why is that? |
| 5 | What do you think is needed for changes in the health of team members to be noticed in time? |  |
| 6 | What conditions can facilitate the implementation of health-oriented leadership? | 1. What challenges are there? |
| 7 | Do you have any further ideas or suggestions for effectively implementing health-oriented leadership in outpatient palliative care? | 1. What would these be? 2. Can you share your ideas with the management team? |
